# Mitochondrial Epigenetics Regulating Inflammation in Cancer and Aging

**DOI:** 10.3389/fcell.2022.929708

**Published:** 2022-07-12

**Authors:** Debmita Chatterjee, Palamou Das, Oishee Chakrabarti

**Affiliations:** ^1^ Biophysics and Structural Genomics Division, Saha Institute of Nuclear Physics, Kolkata, India; ^2^ Homi Bhabha National Institute, Mumbai, India

**Keywords:** mitochondria, epigenetic modifications, inflammation, aging, cancer

## Abstract

Inflammation is a defining factor in disease progression; epigenetic modifications of this first line of defence pathway can affect many physiological and pathological conditions, like aging and tumorigenesis. Inflammageing, one of the hallmarks of aging, represents a chronic, low key but a persistent inflammatory state. Oxidative stress, alterations in mitochondrial DNA (mtDNA) copy number and mis-localized extra-mitochondrial mtDNA are suggested to directly induce various immune response pathways. This could ultimately perturb cellular homeostasis and lead to pathological consequences. Epigenetic remodelling of mtDNA by DNA methylation, post-translational modifications of mtDNA binding proteins and regulation of mitochondrial gene expression by nuclear DNA or mtDNA encoded non-coding RNAs, are suggested to directly correlate with the onset and progression of various types of cancer. Mitochondria are also capable of regulating immune response to various infections and tissue damage by producing pro- or anti-inflammatory signals. This occurs by altering the levels of mitochondrial metabolites and reactive oxygen species (ROS) levels. Since mitochondria are known as the guardians of the inflammatory response, it is plausible that mitochondrial epigenetics might play a pivotal role in inflammation. Hence, this review focuses on the intricate dynamics of epigenetic alterations of inflammation, with emphasis on mitochondria in cancer and aging.

## Introduction

Inflammation, one of the first lines of defence is frequently repurposed from its fundamental role in immune surveillance to a pro-tumorigenic role. Recent studies report that inflammation can aid proliferation of cancer cells and promote tumor microenvironment by selectively blocking anti-tumor immunity (Greten and Grivennikov, 2019). Acute inflammation might be initiated due to several factors, like bacterial or viral infection, autoimmune diseases, obesity, tobacco smoking, asbestos exposure, and excessive alcohol consumption. On the other hand, chronic inflammation has been suggested to be involved in almost all the stages of tumorigenesis. This can further aggravate the phenotype, by generating a pro-tumorigenic inflammatory microenvironment (Grivennikov et al., 2010). Inflammation is hence, considered one of the pivotal factors responsible for predisposition to cancer development (Greten and Grivennikov, 2019). Apart from cancers, chronic or acute inflammation is also strongly associated with age related disorders including atherosclerosis, diabetes, Alzheimer’s disease, rheumatoid arthritis, and aging (Rea et al., 2018). Aging related low grade persistent inflammation is known as ‘senoinflammation’. This is affected by factors, like proinflammatory senescence-associated secretome, inflammasome, ER stress, Toll like Receptors (TLRs), and microRNAs (Chung et al., 2019). Inflammageing, is described as a condition characterized by elevated levels of blood inflammatory markers that signify high susceptibility to chronic morbidity, disability, frailty, and premature death. Some of the plausible etiologies of inflammageing are obesity, altered gut permeability and microbiota composition, cellular senescence, NOD-, LRR- and pyrin domain-containing protein 3 (NLRP3) inflammasome activation, mitochondrial oxidative stress, immune cell dysregulation, genetic predisposition, and chronic infections. Inflammageing can lead to multiple pathological conditions including chronic kidney disease, diabetes mellitus, sarcopenia, depression, dementia as well as cancer (Ferrucci and Fabbri, 2018). Mitochondria, besides being the powerhouse of a cell perform a wide array of functions, like maintenance of calcium homeostasis, orchestration of apoptosis and differentiation (Missiroli et al., 2020). Recent scientific advances reveal that mitochondria actively participate in evoking innate immune and inflammatory responses. Mitochondrial dysfunctions can lead to severe chronic inflammatory disorders (Missiroli et al., 2020).

**FIGURE 1:**
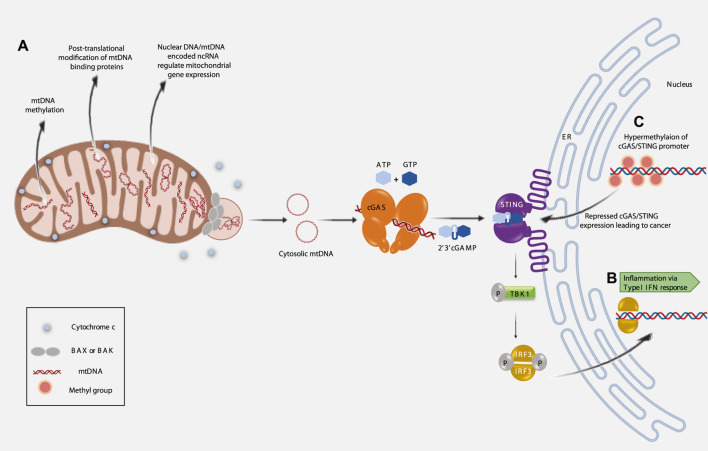
Implications of mitochondrial epigenetics on inflammation, cancer and aging. **(A)**. Three major pathways regulating mitochondrial epigenetics. **(B)**. Extrusion of mtDNA into the cytosol induces inflammation (Type I IFN response) *via* activated cGAS/STING pathway. **(C)**. Epigenetic silencing of cGAS/STING promoter region correlates with cancer prognosis.

It is suggested that epigenetic changes in mitochondria, termed as ‘mitoepigenetics’, are progressively being implicated as heritable changes that can be at the crossroads of several age-related diseases like cardiovascular diseases, osteoarthritis, neurodegenerative diseases and cancers (Coppedè and Stoccoro, 2019). These epigenetic changes include, but are not limited to, alteration in the mitochondrial DNA (mtDNA). Covalent modifications, such as methylation and hydroxymethylation, play a crucial role in altered mtDNA replication and transcription. Post-translational modification of proteins like the mitochondrial transcription factor A (TFAM) is suggested as an essential epigenetic modulator of mtDNA replication and transcription. Post-transcriptional modifications of mitochondrial RNAs (mtRNAs) (like mt-rRNAs, mt-tRNAs and mt-mRNAs) are important epigenetic modulations that affect cellular physiology. mtDNA or nuclear DNA (n-DNA) derived non-coding RNAs (ncRNAs) also play significant roles in the regulation of translation and function of mitochondrial genes (Dong et al., 2020) (Figure 1A). Thus, in this review, we attempt to delineate mitochondrial epigenetic signatures, direct or indirect, which affect inflammation and alter the immune-surveillance mechanism leading to inflammageing, cancer and aging.

### Inflammation and Aging

Mitochondrial dynamics, cellular differentiation and glucose oxidation processes regulate local and systemic inflammation. Mitochondria adapt to oxidative stress by regulating the processes of fission/fusion, optimizing mitochondrial biogenesis, and altering the integrity and copy number of mtDNA (Lee and Wei, 2005; Chen et al., 2018). These mitochondrial processes are similarly affected during oxidative stress associated aging. The activity of the pivotal regulators of mitochondrial biogenesis, like peroxisome proliferator-activated gamma coactivator (PGC)-1α, TFAM, and nuclear respiratory factor 1 (NRF-1), is controlled by post-translational modifications. These modifications are also implicated in regulating mitochondrial metabolism (Skuratovskaia et al., 2021). An increase in Interleukin 6 (IL-6), tumor necrosis factor (TNF)-α, and their receptor levels are detected in aged tissues and cells. IL-6 family cytokines and its receptor complex (with gp130 subunits) have been found to regulate mitochondrial dynamics by decreasing TFAM protein production in liver biopsies of obese patients with and without Type 2 diabetes (Skuratovskaia et al., 2021). Cellular senescence elicits senescence associated secretory phenotype (SASP). This evokes several inflammatory cytokines, chemokines as well as matrix metalloproteases. Aging leads to impaired clearance of senescent cells, thus leading to elevated SASP and chronic inflammation. The mitochondrial dysfunction-associated senescence (MiDAS), can lead to the release and accumulation of mitochondrial components which are recognized as damage-associated molecular patterns (DAMPs). NLRP3 inflammasome identifies DAMPs and promotes its self-oligomerization, leading to the secretion of activated Caspase-1. Activated Caspase-1 further promotes the release of proinflammatory cytokines, including IL-1β and IL-18. Viral infections induce the accumulation and aggregation of signature molecules known as mitochondrial antiviral-signaling proteins (MAVS) on the mitochondrial outer membrane (OMM). This leads to chronic inflammation by the activation of interferon regulatory factor 3 (IRF3) and the NF-κB pathway (Thoudam et al., 2016). Elevated blood serum levels of IL-1 and IL-18 are associated with aging. This indicates that increased secretion of pro-inflammatory cytokines is an early event in aging associated inflammation (Dinarello, 2006). On the contrary, blockade of NLRP3 has been shown to greatly reduce multiple aging associated degenerative changes like insulin resistance, thymic involution, T cell senescence, and bone loss as well as physical and cognitive function decline (Zhu et al., 2021). ROS produced by dysfunctional mitochondria can also trigger an inflammatory response by activating the NF-κB signalling pathway (Ferucci and Fabbri, 2018). Further, the association of cytosolic oxidized mtDNA with NLRP3 has emerged as an essential prerequisite for activation of the inflammasome complex; this results in uncontrolled inflammation as evidenced in several diseases. Furthermore, recent studies have implicated that the increased systemic inflammation observed in aging individuals is associated with increased circulating cytosolic mtDNA. All these, indirectly point towards a role for mitoepigenetics in inflammation and inflammageing (Picca et al., 2018).

### Inflammation and Cancer

Almost 90% of cancers are caused by somatic mutations and environmental factors, barring a few that are associated with germline mutations. These environmental causes and cancer risk factors are mostly associated with some form of chronic inflammation (Multhoff et al., 2012). Viral or bacterial infection induced cancers transform the protective immune inflammation response triggered as the first line of immune defence, into a persistent, low-grade chronic inflammation. This generates a beneficial microenvironment for the tumor to sustain and proliferate. A low mtDNA copy number is associated with a heightened inflammatory response; it triggers elevated levels of hs-CRP, IL-6, fibrinogen, and increases white blood cell count (Wu et al., 2017). Many cellular responses involved in cancer have been implicated to interact with the signal transducer and activator of transcription 3 (STAT3) protein, a transcription factor known to mediate cytokine signalling. This, in turn, induces sustained autophosphorylation, maintenance of enhanced proliferation and upregulation of antiapoptotic BCL-xL and Cyclin-D. Inflammation in general is a self-restricting phenomenon with a balance between the anti-inflammatory and proinflammatory cytokines. However, in presence of tumorigenic insults, the proinflammatory cytokines over-ride the anti-inflammatory cascade and lead to a chronic inflammatory state, comprising cytokines that propagate tumorigenic growth. Interestingly, the inflammatory signalling pathway comprising IL-6 and STAT3 molecules have been implicated in stomach, colorectal, bladder and lung cancers (Coussens and Werb, 2002). Inflammatory factors, like cytokines, chemokines, growth factors, inflammasomes and inflammatory metabolites have emerged as regulators of tumorigenicity. They do so by modulating multiple signalling pathways, such as nuclear factor kappa B (NF-kB), Janus kinase/signal transducers and activators of transcription (JAK-STAT), toll-like receptor (TLR) pathways, cGAS/STING, and mitogen-activated protein kinase (MAPK) pathways (Zhao et al., 2011).

### Inflammation and Epigenetics

Innate immune responses, elicited during tissue damage or microbial infection are known to induce inflammation (Akira et al., 2006; Brennan et al., 2015). The presence of cytosolic DNA, like microbial DNA or part of nuclear DNA (that has escaped from the nucleus), can trigger innate immunity. Under such conditions, two proteins play essential roles in eliciting innate immune responses—these are, 1) cyclic GMP-AMP synthase (cGAS), a cytosolic DNA sensor and 2) stimulator of interferon genes (STING), an ER resident protein (Sun et al., 2013). It is now, well established, that mtDNA released into the cytosol can bind cGAS (McArthur et al., 2018; Riley et al., 2018; Kim et al., 2019). The concerted activity of cGAS and activated STING initiates a signalling cascade that culminates in the transcription of Interferon stimulated genes (ISGs) (McArthur et al., 2018; Riley et al., 2018; Kim et al., 2019; Zhang et al., 2019). Thus, the presence of cytosolic mtDNA can elicit inflammation via an innate immune response (Figure 1B).

Interestingly, it has been observed that cGAS and/or STING expression is decreased in various cancers, like—colon cancer and melanoma (Xia et al., 2016a; Xia et al., 2016b). Reduced cGAS/STING expression corresponds with poor survival in lung and gastric cancer patients (Song et al., 2017; Yang et al., 2017). One of the reasons, for the loss of cGAS-STING signalling is suggested to be the epigenetic silencing of cGAS/STING promoter regions (Konno et al., 2018). Hypermethylation of cGAS/STING promoters contributes to the transcriptional silencing and perturbed STING signalling function is implicated in various cancers (Konno et al., 2018; Falahat et al., 2021) (Figure 1C). Hence, the interconnectivity between all these factors opens up new and important avenues for future research as this would help establish their therapeutic potential.

### Mitochondrial Epigenetics

Mitochondrial epigenetics remains less understood primarily due to the lack of classical epigenetic regulators and substrates for mtDNA. However, epigenetic regulation of mtDNA may be potentiated by post-translational modification on mtDNA interacting proteins. Mitochondrial metabolites can also serve as substrates for epigenetic modifications (Weise and Bannister, 2020). Circular mtDNA (16,569 base pairs) comprises one purine rich heavy strand and the complementary light strand is pyrimidine rich (Asin-Cayuela and Gustafsson, 2007). There is another linear strand, 7S DNA that forms the displacement loop or D-loop; however, its presence is not ubiquitous through all cell types and organisms (Nicholls and Minczuk, 2014). mtDNA is maternally inherited and intron-less (Iacobazzi et al., 2013); it also lacks histone protein. Thus, unlike nuclear DNA, epigenetic regulation of mtDNA is methylation-dependent.

Classically, during DNA methylation, a methyl group is added from S-adenosyl-methionine (SAM) to DNA bases cytosine (C) or adenine (A) by DNA methyltransferase (DNMT) enzymes. DNA methylation is usually observed at the CpG islands in the promoter region. mtDNA methylation has been a debatable subject. It was believed that mtDNA would not get methylated as mitochondria are inaccessible to methylase and mtDNA is not complexed with histones (Iacobazzi et al., 2013). However, reports suggest that mtDNA methylation does occur in a non-random manner. Due to its small size, CpG islands are absent, but 3%–5% CpG dinucleotides of mtDNA are found to be methylated (Pollack et al., 1984). The presence of DNMT1, targeted to mitochondria (mtDNMT1) further, emphasizes the methylation event of mtDNA. mtDNMT1 is a nuclear encoded protein, which consists of a mitochondrial targeted sequence (MTS) upstream of the translation start site (Shock et al., 2011). mtDNMT1 is detected on the outer mitochondrial membrane in adult neurological tissues, heart and skeletal muscles (Wong et al., 2013). Varying expression levels of mtDNMT1 in cells is shown to affect gene expression pattern -like when mtDNMT1 is overexpressed, the protein coding gene from the light strand promoter (LSP) MT-ND6, gets significantly downregulated (Shock et al., 2011). However, in the same condition, MT-ND1 is upregulated from heavy strand promoter (HSP) without affecting MT-ATP6 or MT-CO1 (Shock et al., 2011). DNA methylation, is mostly observed in D- loop region of mtDNA comprising both HSP and LSP promoter elements. However, the exact mechanism by which mtDNA gets methylated or demethylated remains potentially elusive, till date. Identifying all the participating enzymes would be the first step in that direction. ALKBH1, a demethylase is reported to affect oxidative phosphorylation in mitochondria (Koh et al., 2018). The presence of ten-eleven translocation (TET) 1 and 2 suggests oxygen mediated demethylation in mtDNA (Dzitoyeva et al., 2012). Demethylation of cytosine residues can also be achieved by deamination. Identification of APOBEC3 (Apolipoprotein B mRNA editing enzyme catalytic polypeptide-like 3) in mitochondria further suggests epigenetic regulation of mtDNA (Wakae et al., 2018).

In the nucleus, the epigenetic function is controlled through post-translational modifications of histone proteins. As mentioned earlier, mtDNA lacks histone proteins, however, the DNA binding proteins could serve as targets for post-translational modifications. mtDNA is present in the nucleoids, which are membrane-less pseudo-compartments in mitochondrial matrix comprising nucleoprotein complexes. It is reported that, 63% of all proteins localized within the mitochondria consist of lysine acetylation sites. Numerous phosphorylation sites are also suggested to be present in those proteins (Zhao et al., 2011). One of the most studied nucleoid associated proteins, TFAM, is involved in mtDNA compaction and transcription. TFAM can be post-translationally modified by acetylation, O-linked glycosylation and phosphorylation (Suarez et al., 2008; Lu et al., 2013; King et al., 2018). Being a member of the high mobility group (HMG) protein, TFAM binds mtDNA co-operatively as a homodimer (Kaufman and Van Houten, 2017). Alteration in the binding affinity of TFAM, affects the mtDNA replication and transcription rates. When the dimer/monomer ratio of TFAM increases, heavy strand replication is stopped, and transcription starts. Also, mtDNA transcription is halted when TFAM/mtDNA ratio is high (Audano et al., 2014). Phosphorylation of HMG1 inhibits the binding of TFAM to mtDNA, preventing activation of transcription (Lu et al., 2013). Other nucleoid associated proteins also have phosphorylation sites, like mtSSB (mitochondrial single strand binding protein) and POLG (DNA polymerase gamma) (Matsuoka et al., 2007; Zhou et al., 2013). But the accurate mechanism of epigenetic control through post- translational modification is yet to be completely unravelled.

Further, nuclear DNA and mtDNA encoded lncRNAs can regulate mitochondrial gene expression. mtDNA encodes for three such lncRNAs — ND5, ND6, and CYB. Nuclear DNA encoded RNaseP complex can control the expression of these lncRNAs. These three lncRNAs, are capable of forming intermolecular duplexes with their functional counterparts, and thus can regulate their expression (Rackham et al., 2011). Another example of mtlncRNAs containing MDL1 (mitochondrial D-loop 1), which spans the anti-sense region of tRNApro and mitochondrial D-loop. The functional importance of mtlncRNAs, however, remains elusive. D-loop is slowly emerging as one of the most essential components of mtDNA for epigenetic regulation. MDL1 and its anti-sense could also participate in epigenetic regulation of mtDNA significantly (Gao et al., 2018). RNA processing endoribonuclease (RMRP) is a lncRNA encoded in nucleus, but transported to mitochondria. It can modify mtDNA replication and transcription (Wang et al., 2010; Noh et al., 2016).

Besides lncRNAs, small non coding RNAs also play a crucial role in the epigenetic regulation mechanism. Mitochondrial microRNAs or mito-microRNAs (mitomiRs) are single stranded 17–25 bp long RNA molecules, either encoded by nuclear DNA and transported to mitochondria or transcribed from mtDNA (Bandiera et al., 2011; Sripada et al., 2012; Ro et al., 2013). Complementary base pairing between miR-2392 and mtDNA in an argonaute-2 (AGO-2) dependent manner prevents mtDNA transcription partially and affects OXPHOS protein expression. miR-181C targets the 3’ end of MT-CO1 mRNA to repress its expression (Das et al., 2012; Das et al., 2014). Translocation of miR-1 and miR1a-3p causes upregulation of MT-CO1 and MT-ND1 (He et al., 2012; Zhang et al., 2014).

### Mitochondrial Epigenetics in Inflammation

As already indicated, mitochondrial epigenetics to date is rather less explored. Hence, it’s implication in various signalling pathways contributing to varied disease phenotypes are being investigated only recently. Phosphorylation of TFAM by cAMP-dependent protein kinase in mitochondria, within its HMG box 1 leads to impaired ability of binding of TFAM to DNA and hence decreased transcription of mtDNA (Lu et al., 2013). Alteration of mtDNA copy number directly regulates inflammatory response. Hence, it is plausible to hypothesize that TFAM/mtDNA/interleukin axis plays a pivotal role in diseases like osteoarthritis and neurodegeneration (Kang et al., 2018; Zhan et al., 2020). Among the post-translational modifications, ubiquitination of TFAM has been implicated in the disease prognosis of diabetic retinopathy (Santos et al., 2014). Alterations in the activity of the mtDNMT1 have been indicated in modulating methylation profiles and transcription efficiency of various signalling pathways including inflammation and angiogenesis. These are also important in several common age-related pathologies and cancer (Shock et al., 2011; Atilano et al., 2015). Hypoxia is known to turn on the hypoxia-responsive transcription factors including peroxisome proliferator-activated receptor gamma coactivator 1 alpha (PGC1α) and nuclear respiratory factor 1 (NRF1). These further upregulate mtDNMT1 activity and cause hypermethylation of mtDNA. This leads to repressing gene expression from the light strand promoter during vascular oxidative stress. Recently mtDNA methylation has emerged as a novel non-invasive epigenetic biomarker and is implicated in the etiology of cardiovascular diseases, where increased mtDNA methylation of genes encoding for cytochrome c oxidases, tRNA leucine 1 as well as genes involved in ATP synthesis have been reported (Mohammed et al., 2020). Hypermethylation of mtDNA ND-6 has been implicated in non-alcoholic fatty liver disease and is suggested to be strongly associated with steatohepatitic condition. Steatohepatitis is an aggressive form of liver disease characterized by liver inflammation that ultimately progresses to cirrhosis and liver failure. Hence, the association of epigenetically modified mtND-6 in steatohepatitis could highlight the importance of mitoepigenetics in inflammation and prognosis of certain diseases (Pirola et al., 2013). Further, mitomiRs, a subset of miRNAs, are potential epigenetic regulators of the mitochondria. They affect some of the major mitochondrial functions, like maintenance of membrane potential and electron transport chain (ETC). miR-107 is known to affect the oxidative pathway of mitochondria and its reduction leads to a decrease in mitochondrial volume and altered cristae. It causes mitochondrial dysregulation due to a reduction in mitochondrial membrane potential and ETC activity by decreasing the protein levels of complexes 1,3,4, and 5 (John et al., 2020). miR-125b is implicated in neural cell apoptosis by switching the balance between BAX and BCL-2 towards an apoptotic fate. BCL-2 and BAX can, in turn, regulate mitochondrial membrane permeability by inducing transition pore formation and release of Cytochrome c. This suggests an antitumorigenic effect of mitoepigenetics brought about by enhancing apoptosis. Furthermore, miR-125b is known to negatively regulate IL1β-induced inflammatory genes by targeting the TRAF6-mediated MAPKs and NF-κB signalling in human osteoarthritic chondrocytes (Rasheed et al., 2019).

Inflammation is indirectly regulated by mitochondrial epigenetics *via* altered ROS production and mitochondrial metabolism. These, in turn, affect the known direct players of mitoepigenetics like methylation of DNA, mtDNMT1 activity, release of mtDNA, and TFAM expression. Mitochondrial ROS levels affect DNA methylation (Kietzmann et al., 2017). ROS can directly convert 5-methylcytosine to 5-hydroxymethylcytosine, thereby, blocking the activity of DNMT1. This leads to global hypomethylation. ROS can also oxidize guanosine to 8-oxo-20-deoxyguanosine (8-oxodG) and inhibit the methylation of adjacent cytosine. This can further contribute to the global hypomethylation of DNA. Evidence shows that the formation of 8-oxodG promotes the transcription of TNF-α responsive pro-inflammatory genes. 8-oxodG is also capable of interacting with HIF1α and negatively modulates its binding with the VEGF promoter. This results in impaired angiogenesis. In line with these observations, two recent meta-analyses have shown that high levels of 8-oxodG are associated with atherosclerotic vascular disease and predicts the eventual disease prognosis (Hooten et al., 2012; Carracedo et al., 2020). High ROS levels also influence both repressive (H3K9me2/3 and H3K27me3) and active histone marks (H3K4me2/3). Hence, it may as well be proposed that mitochondrial metabolism and DNA methylation go hand-in-hand (Audia and Campbell, 2016; Lopes, 2020).

### Mitoepigenetics in Cancer

Silencing the key regulator of mtDNA, TFAM, leads to a pro-tumorigenic microenvironment (Araujo et al., 2018). This favours metabolic reprogramming towards aerobic glycolysis—as is suggested by decreased respiratory capacity coupled with increased lactate production. Secondly, enhanced ERK1/2-Akt-mTORC-S6 signalling activity leads to enhanced cell growth, metastasis and chemoresistance. On the other hand, increased TFAM expression leads to a significant reversal of these phenotypic changes (Hsieh et al., 2021). Cell lines like those derived from gynaecological origin (ovarian cancer) are known to have upregulated TFAM; this positively correlates with cell proliferation, colony formation, migration, and invasion. It supports a protumorigenic phenotype (Hu et al., 2020). MitomiRs have been implicated to regulate various important tumorigenic phenotypes like, alteration of mitochondrial bioenergetics, invasion, and angiogenesis. miR-126 is known to alter mitochondrial energy metabolism by reducing mitochondrial respiration and promoting glycolysis. This is executed *via* IRS1 associated modulation of ATP-citrate lyase deregulation; this leads to suppression of the malignant mesothelioma tumor phenotype. An increase in ATP and citrate production leads to reduced Akt signalling and cytosolic sequestration of Forkhead box O1 (FoxO1). This leads to reduced expression of downstream genes involved in gluconeogenesis and defence against oxidative stress. miR-126 is suggested to play an important regulatory role in multiple human cancers, like breast, lung, gastric cancers, melanoma and acute leukaemia (Tomasetti et al., 2012).

Among the several oncogenic stimuli, hypoxia has been reported to alter mitomiR expression (Giuliani et al., 2018). Under conditions of hypoxia, both normal and transformed cells have elevated levels of miR-210 expression, suggesting its role in an adaptive response to this stress (Puisségur et al., 2011). It is now believed that elevated miR-210 expression represents hypoxia gene signatures in tumor tissues like those of breast, head and neck cancers. miR-210 can regulate various signalling mechanisms, like those involved in the cell cycle, survival, differentiation, angiogenesis, and metabolism. Over-expression of miR-210 is further reported in lung cancer derived cell line, A549; thus, suggesting the role of mitomiRs in lung cancer (Grosso et al., 2013; Qin et al., 2014). Another important mitomiR identified to be involved in tumor progression is miR-200 (Korpal and Kang, 2008). One of the prime miR-200 targets is TFAM, which has been implicated both in regulating mitochondrial biogenesis and inflammation. TFAM has been described as a functional target of miR-200 in breast cancer cells. Since TFAM is a transcription factor, its activity is required for mtDNA replication, transcription and maintenance. An alteration in the quality control of mtDNA severely affects the inflammation process. TFAM has also been implicated as a primary architectural protein of the mitochondrial genome by packaging mtDNA. In addition, TFAM expression has been reported to be involved in tumor progression, cancer cell growth, and chemoresistance (Rencelj et al., 2021). Further, the reduced mtDNA copy number is associated with several aggressive phenotypes, like the onset of apoptosis, metabolic shift towards glycolysis, and increased invasiveness in various human cancers (Wu et al., 2017). All these taken together suggest that mitochondria and their epigenetic modifications are closely associated with the tumorigenic phenotypes of invasion, metastasis and chemoresistance in many types of cancers.

### Mitoepigenetics in Aging

Mitochondrial dysfunction is implicated at the core of the aging process; this mainly comprises mtDNA mutations, impaired respiratory chain functions and elevated ROS production (Trifunovic and Larsson, 2008). Altered mtDNA methylation can lead to enhanced ROS production. ROS is a known messenger of the inflammatory cytokine signalling pathway. Taken together, it is plausible to hypothesize a complex inter relationship between the three processes of mitoepigenetics, inflammation and aging. Experiments on mtDNA methylation within the 12S ribosomal RNA gene has shown that hypomethylation of two CpG sites (M1215 and M1313) have a direct correlation with age. This suggests that mtDNA methylation could be an epigenetic marker of aging (Mawlood et al., 2016). Further, decreased levels of 5-hydroxymethylcytosine on mtDNA, but not 5-methylcytosine, is detected in the frontal cortex of aging mice (Dzitoyeva et al., 2012). Reduced 5-hydroxymethylcytosine correlates with increased mRNA levels of ND2, ND4, ND4L, ND5 and ND6 regions of the mitochondrial D-loop. This could in turn be due to the downregulation of DNMT1 and upregulation of TET2 in the mitochondria of the frontal cortex of aging mice (Dzitoyeva et al., 2012). Higher mitochondrial 12S rRNA gene (RNR1) methylation corresponds with increased mortality risk—this hence suggests the importance of mitochondrial epigenetics in aging and survival (D’Aquila et al., 2015). Recently, decreased global methylation level of both mtDNA strands is suggested to be associated with aging (Dou et al., 2019). mtDNA methylation is implicated to play a pivotal role in aging *via* the regulation of mitochondrial gene expression (Cao et al., 2021). Again, methylation profiling studies of humans over a wide age range have revealed that missense mutations in the six-transmembrane epithelial antigen of the prostate-2 (STEAP2) gene are associated with the maintenance of homeostasis of metal ions. These metal ions (iron and copper) are known to play a role in the proper functioning of the ETC. This would lead to further complications like ROS mediated anomalies and impaired DNA damage—all ultimately culminating in an aggravated ‘senile’ state (Hannum et al., 2013). The conserved histone lysine demethylases, jmjd-1.2/PHF8 and jmjd-3.1/JMJD3 are reported to be positive regulators of lifespan. Their presence across species suggests an evolutionarily conserved mitoepigenetic mechanism (Merkwirth et al., 2016). Since aging and mitochondrial dysfunction are interdependent, it is rational to hypothesize that mitochondrial stress induced methylation marks and associated downstream signalling mechanism might potentially contribute to the aging process. It has been found that mitochondrial stress response activation is associated with the di-methylation of histone H3K9 through the activity of the histone methyltransferase met-2 and the nuclear co-factor lin-6. This leads to global gene silencing, though there are portions of the chromatin which open up due to the binding of canonical stress responsive factors, like DVE-1. A metabolic stress response specific gene expression signature negatively modulates the aging phenotype, ultimately leading to an extension of lifespan (Tian et al., 2016).

In some age-related neurodegenerative diseases, mtDNA methylation is found to be critically important. Evidence shows increased detection of 5-methylcytosine levels in the mtDNA D-loop region in Alzheimer disease-related pathology. Further, lower 5-methylcytosine levels in mtDNA D-loop region are also detected in patient samples positive for Parkinson’s disease (Blanch et al., 2016). Experiments in transgenic mice have shown decreased D-loop methylation and elevated RNR1 methylation in the hippocampus region (Xu et al., 2019). Patients with Down syndrome are reported to have decreased levels of the methyl group donor SAM (S-Adenosyl Methionine), correlating with hypomethylation of mtDNA (Infantino et al., 2011). All these taken together suggest a close correlation between mitoepigenetics and the process of aging.

## Conclusion

**FIGURE 2:**
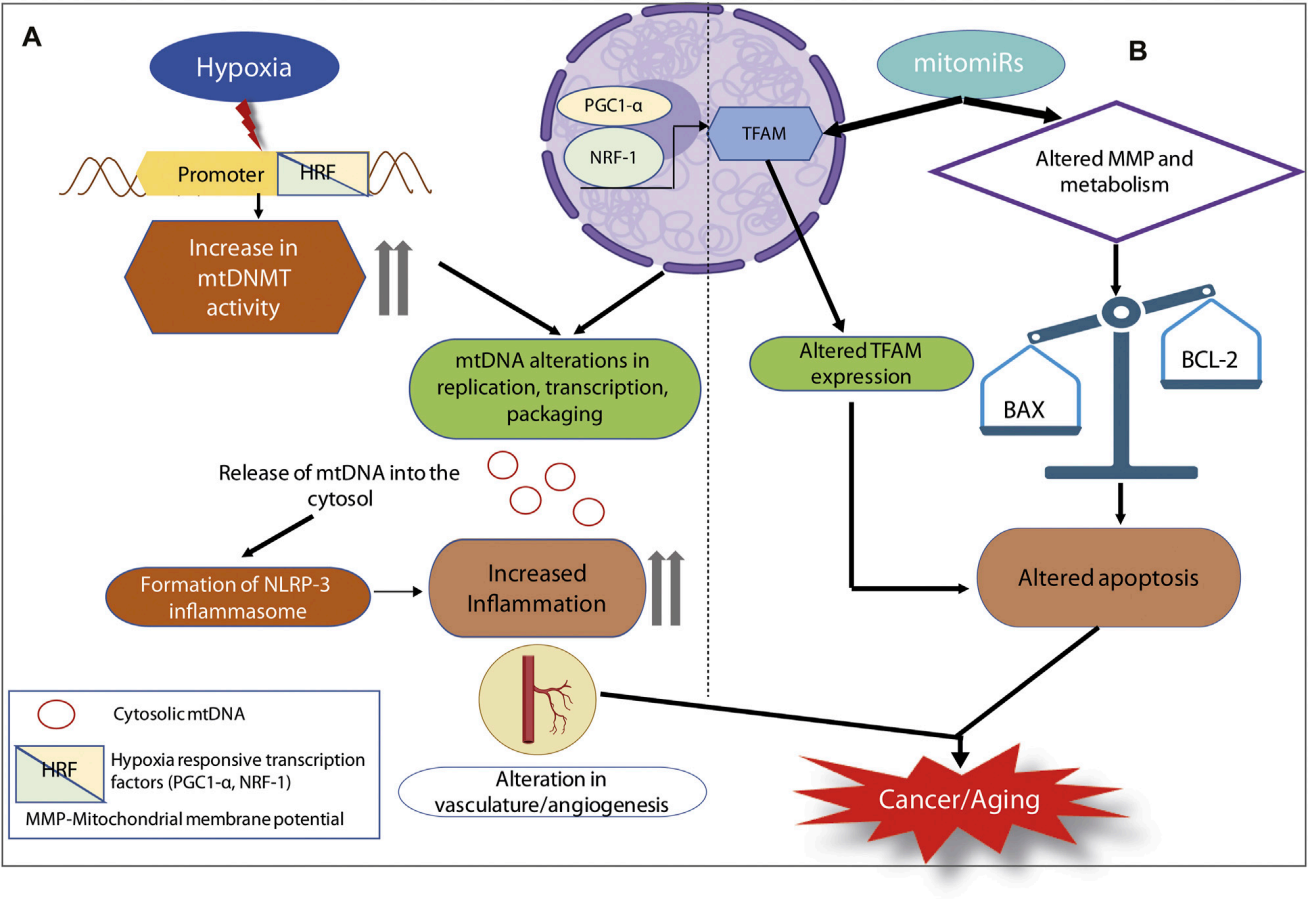
Mitoepigenetic regulation of inflammation in cancer or aging. **(A)** Hypoxia as an oncogenic stimulus, it turns on the HRFs. HRFs alter mtDNMT activity as well as the expression of TFAM. They can combinatorially lead to several mtDNA alterations and the release of mtDNA into the cytosol. Extrusion of cytosolic mtDNA can trigger the formation of NLRP3 inflammosome, elevated inflammation culminating in cancer or aging. **(B)** mitomiRs are capable of altering TFAM expression as well as mitochondrial membrane potential and metabolic status. These two pathways can converge on the apoptotic fate of cell, thereby leading to cancer or aging.

Inflammation is regulated by several factors. Mitochondria have now emerged as central in innate immunity, inflammatory responses, aging and cancer. Likewise, mitochondrial epigenetics, though less understood is fast gaining significance as a potential regulator of inflammation and an important contributing factor for physiological and pathological conditions, like aging, neurodegenerative diseases and cancer (Liu et al., 2016; Iske et al., 2020). It is now well understood that mitochondrial epigenetics reaches beyond the confines of classical epigenetic signatures as these organelles lack histones and the conventional CpG islands. Studies have reported that methylation and demethylation of mtDNA could bring about the repression of downstream genes like mtND-6, mt-ATP6 and mt-CO1 (Stoccoro and Coppedè, 2021). Altered expression of these genes leads to differences in mitochondrial metabolism (like glucose metabolism). This would ultimately regulate mitochondrial antiviral signalling protein (MAVS) and result in cGAS/STING mediated immune dysfunction (Zou et al., 2021). Further, the presence of cytosolic mtDNA can trigger inflammation *via* cGAS/STING pathway (Bahat et al., 2021). mtDNA dysfunction due to changes in the copy number, altered compaction, deregulated transcription, or extrusion into the cytosol, is the leading driver for NLRP3 mediated inflammosome formation (Zhong et al., 2018). Post- translational modifications of TFAM, mtDNMT1 activity and hypoxia contribute to changes in mtDNA. This culminates in mtDNA induced inflammation, as is reported in diseases like lung cancer, osteoarthritis, neurodegeneration etc (Nakayama and Otsu, 2018; Iske et al., 2020). Altered mitochondrial DNA methylation, alongwith the deregulated balance between methylases and demethylases is fast emerging as epigenetic markers of aging (Mawlood et al., 2016). mtDNA methylation that affects the expression of certain genes responsible for the maintenance of metal ion (iron, copper) homeostasis affects “senoinflammation” and hence could also regulate aging. Also, altered mtDNA methylation is detected in multiple age-related neurological disorders. Hypoxia, hypoxia responsive factors like PGC1α or NRF1, can alter the mtDNMT1 activity, bring out changes in methylation of mtDNA, the interleukin axis (that releases proinflammatory cytokines like TNF-α), and result in inflammation. All these can eventually have pathological outcomes. Differential expression of mitomiRs might regulate TFAM expression and alter the mitochondrial membrane potential and metabolism. These lead to cellular changes, like skewing the balance between pro-apoptotic and anti-apoptotic proteins, and altered interleukin signalling. Mitoepigenetic regulation of inflammation, tissue remodelling, cellular differentiation, enhanced vasculature and angiogenesis would culminate in a pro-cancerous phenotype (Figure 2). Mitoepigenetics in its many forms is at the crossroad of immune signalling and inflammation; this modulates the physiological process of aging and affects the pathology of various cancers. Thus, strategies aimed at compensating for changes brought about by mitoepigenetics like restoration of dysfunctional mtDNA or TFAM activity might emerge as promising preventive and therapeutic interventions for pathological conditions occurring due to exacerbated inflammation.
